# New Inventions

**Published:** 1895-03

**Authors:** 


### NEW INVENTIONS.

Automatic watering-trough for stalls.

Salt mouth medicine bottle-stopper, with scoop attached.

A removable bridle blind, making an open or closed bridle at will.

A horse-muzzle in two hinged sections held together with a spring fastening.

Patent hitching-strap with ring, spring tongue-snap and coiled spring attachment, to make immovable to post or place hitched.

Farrier's shoeing stand, enabling the shoer to sit and the horse's foot to be supported when the nails are being driven and clinched.

A heel-padded horseshoe, the yielding material being placed between the heel of the shoe and the foot, secured to the shoe through holes in the metal.

